# NSCLC Mutated Isoforms of CCDC6 Affect the Intracellular Distribution of the Wild Type Protein Promoting Cisplatinum Resistance and PARP Inhibitors Sensitivity in Lung Cancer Cells

**DOI:** 10.3390/cancers12010044

**Published:** 2019-12-21

**Authors:** Aniello Cerrato, Francesco Morra, Imma Di Domenico, Angela Celetti

**Affiliations:** Institute for the Experimental Endocrinology and Oncology “Gaetano Salvatore”, Italian National Council of Research, Via S. Pansini 5, 80131 Naples, Italyimma.didomenico1@gmail.com (I.D.D.)

**Keywords:** CCDC6, cell-cycle-checkpoint, homologous-recombination, dominant-negative, DNA-damage-repair, biomarker, BRCAlike, synthetic-lethal, targeted-therapy, PARP-1-inhibitors-sensitivity

## Abstract

CCDC6 is implicated in cell cycle checkpoints and DNA damage repair by homologous recombination (HR). In NSCLC, CCDC6 is barely expressed in about 30% of patients and CCDC6 gene rearrangements with RET and ROS kinases are detected in about 1% of patients. Recently, CCDC6 point-mutations naming E227K, S351Y, N394Y, and T462A have been identified in primary NSCLC. In this work, we analyze the effects exerted by the CCDC6 mutated isoforms on lung cancer cells. By pull-down experiments and immunofluorescence, we evaluated the biochemical and morphological effects of CCDC6 lung-mutants on the CCDC6 wild type protein. By using two HR-reporter assays, we analyzed the effect of CCDC6 lung-mutants in perturbing CCDC6 physiology in the HR process. Finally, by cell-titer assay, we evaluated the response to the treatment with different drugs in lung cancer cells expressing CCDC6 mutants. This work shows that the CCDC6 mutated and truncated isoforms, identified so far in NSCLC, affected the intracellular distribution of the wild type protein and impaired the CCDC6 function in the HR process, ultimately inducing cisplatinum resistance and PARP-inhibitors sensitivity in lung cancer cells. The identification of selected molecular alterations involving CCDC6 gene product might define predictive biomarkers for personalized treatment in NSCLC.

## 1. Introduction

Coiled Coil Domain Containing 6 (CCDC6) gene product is ubiquitously expressed and target substrate of several serine/treonine kinases named ERK1/2, ATM, CDK1, and GSK3β, which, depending on the intracellular signaling, modulate the stability and the intracellular distribution of the protein [1,2,3,4,5,6,7]. In the nucleus CCDC6 exerts a main action in the process of DNA homologous recombination (HR) for the repair of DNA double strand breaks (DSB) and for the cell mitotic entry. Upon DNA damage, CCDC6 is phosphorylated by ATM and, by interacting with the H2AX histone-phosphatase PP4c, CCDC6 promotes the stability of DNA repairing foci which are marked by the phosphorylated histone H2AX at the S139 and by RAD51 protein [8,9]. 

In vitro evidence indicates that the silencing of CCDC6 in cancer cells releases the PP4c phosphatase-activity, lowers the levels of phospho H2AX^S139^ and reduces the number of DNA repairing foci upon exposure to DNA damage inducers. The silencing of CCDC6 in cancer cells exposed to DNA damage also decreases the levels of the G2-checkpoint phospho-protein Chk1 and shortens the G2 phase, allowing a premature entrance into the mitosis [9,10]. 

Cancer cells carrying HR DNA repair molecular alteration, like BRCA1/2 defects, are selective target of the PARP inhibition, resulting in a synthetic lethal phenotype. Indeed, cancer cells defective for CCDC6 show an impaired HR process and switch to the non-homologous end joining process (NHEJ) to repair their DNA. As the NHEJ process is prone to errors, the cancer cells defective for CCDC6 show tolerance to the DNA damage induced by several mutagens, including cis-platinum. Furthermore, the switch to the NHEJ repair process that involves the poly (ADP-ribose) polymerase enzymes (PARP1/2) makes the CCDC6 defective cells sensitive to PARP inhibition because of a synthetic lethal effect [11].

In several in vitro cancer cell systems CCDC6 protein levels have been correlated with cancer cell tolerance to DNA damage, resistance to cisplatinum and sensitivity to poly (ADP-ribose) polymerase inhibitors (PARPi) [4,11,12,13,14,15,16]. 

To date, besides the first CCDC6 fusion with the tyrosine kinase RET identified in papillary thyroid cancer [17,18], additional CCDC6 fusions have been reported with PDGFRb [19,20], PTEN [21], FGFR2 [22,23] ANK3 [24], and UBE2D1 [25] and with other genes in different tumor types [26].

Following the fusions, the recombinant oncogenes always include, at least in part, the CCDC6 coiled-coil region that is able to induce protein dimers [27,28]. Interestingly, in the case of CCDC6/RET, the first 101 amminoacid (aa) of CCDC6 involved in the fusion can form heterodimers with wild type protein of CCDC6 and impair its physiological function [1]. 

In some cases, cancer cells carrying the rearranged allele of CCDC6 also show the loss of the unrearranged allele which is suggestive of a suppressive role of CCDC6 in tumor progression [29]. 

In NSCLC, the CCDC6 involvement can be ascribed both to the low levels of the protein expression, as reported in 30% of patients, and correlated with tumor progression and bad prognosis (OS and DFS) [16], and to the fusion of the first CCDC6 101 aa with the RET and ROS1 kinases, as detected in about 1% of NSCLC patients. The identification of CCDC6 fusions to other partners (LIP I, CTNNA3, and KITLG) in this type of tumor have been also reported [26,30,31,32,33,34].

Recently, four missense mutations—namely E227K, S351Y, N394Y, and T462A have been identified in the CCDC6 gene sequence in primary NSCLC and are in need of functional characterization (https://cancer.sanger.ac.uk/cosmic), [35]. These missense mutations have been detected in 0.42% of lung samples, and overall represent the 40% of the CCDC6 gene alterations reported in lung, each of them with a pathogenic score higher than 0.9 [36].

Here, we carried out a functional characterization of the four point mutations identified so far in NSCLC, and of the truncated 1–101 isoform of CCDC6, demonstrating their role in the process of DNA damage repair and suggesting them as possible target of PARPi treatment in combination with conventional chemotherapy. 

Given the high incidence of NSCLC in the population and the failure of the conventional anti-cancer therapies for this tumor type, the new molecular alterations that we have characterized can help to define a more precise approach for lung cancer therapy [37].

## 2. Results

### 2.1. CCDC6 Lung-Mutants Form Heterodimers with the Wild Type Protein Shaping Its Intracellular Distribution

**Figure 1:**
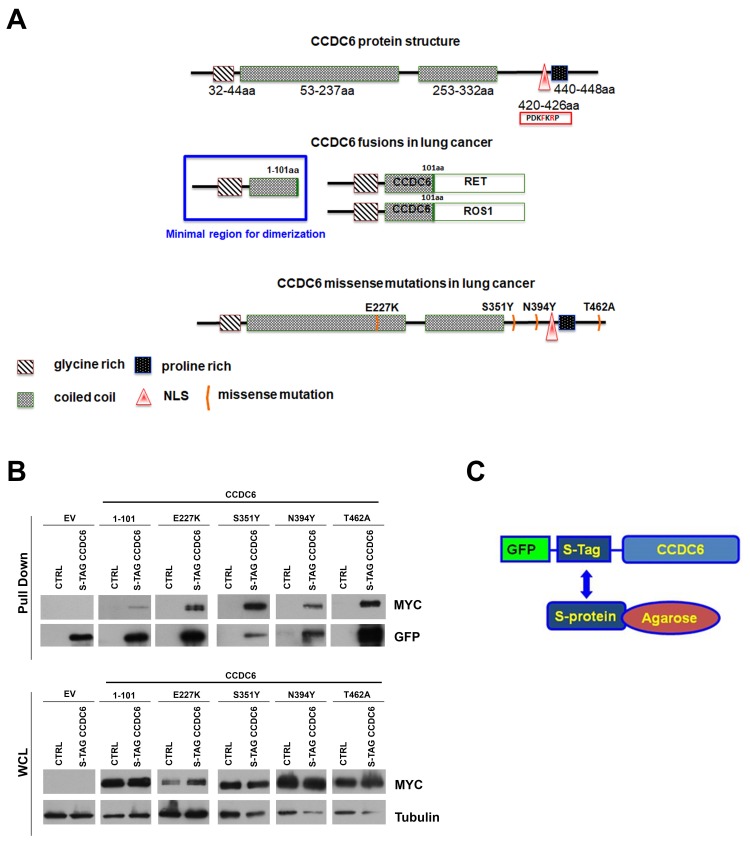
CCDC6 lung-mutants form heterodimers with the CCDC6 wild type protein (**A**) Top: CCDC6 protein diagram showing a glycine rich domain at the NH2 terminus, an extensive coiled coil domain, and a proline rich domain at the COOH terminus. The red triangle in the diagram indicates the nuclear location site (NLS) with the relative sequence (420–426 aa), as reported in the red box. Middle: CCDC6 protein diagram showing the CCDC6 fusions in NSCLC. Bottom: CCDC6 missense mutations identified so far in lung cancer. (**B**) S-tag pull down of HeLa-Kyoto S-tag-GFP-CCDC6 overexpressing the empty vector (EV) or the myc-tagged CCDC6 mutated or truncated isoforms were analyzed by SDS-PAGE and immunoblotted with the anti-myc and anti-GFP antibodies, as shown. The immunoblots of the whole cell lysates (WCL) are shown at the bottom of the panels. (**C**) Schematic representation of the S-Tag-GFP-CCDC6 construct. Results are representative of at least three independent experiments.

Beside the reduced levels of CCDC6 protein expression and the CCDC6 fusions with different kinases, four missense mutations of CCDC6 have been identified so far in NSCLC (Figure 1A). 

The mutated isoforms E227K, S351Y, N394Y, and T462A of CCDC6 tagged with a myc-epitope were expressed in Hela Kyoto S-tag-GFP-CCDC6 cells in order to characterize their function. The missense mutations inserted in the CCDC6 sequence, with the exception of the E227K, are not predicted to affect the coiled-coil structure of the CCDC6 protein (aa 53–237 and aa 253–332), that is necessary for the homodimerization of the CCDC6 wild type protein [4]. Then, by pooling down the S-tag-GFP-CCDC6 protein with the S-agarose beads, we isolated the hetero-complex of each of the CCDC6 mutants with the CCDC6 wild type protein, as the myc-tagged CCDC6 mutants were able to interact and form heterodimers with the S-tagged GFP-CCDC6 wild type protein stably expressed in Hela Kyoto cells (Figure 1B,C; Figures S1 and S4). The myc-CCDC6^1–101^ truncated isoform, corresponding to the CCDC6 region fused to the kinases, was also able to form hetero-complex with the S-tag-GFP-CCDC6 wild type (Figure 1B, second lane).

**Figure 2:**
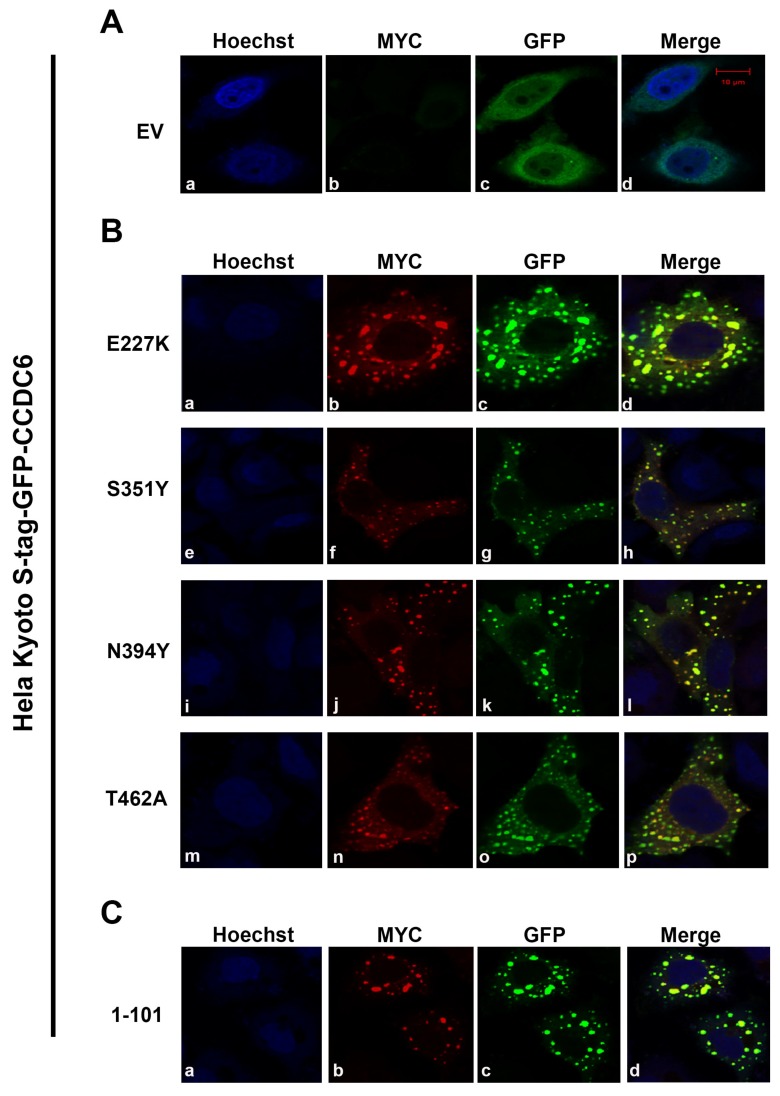
CCDC6 lung-mutants shape the intracellular distribution of the CCDC6 wild type protein. (**A**) In HeLa Kyoto S-tag-GFP-CCDC6 transfected with a pcDNA4ToAmyc-his empty vector the GFP-CCDC6 protein is detected in the cytosol and in the nucleus (**c**), also in the counterstained nuclei (**d**); (**B**) in HeLa Kyoto S-tag-GFP-CCDC6, the tranfected myc-CCDC6 mutated isoforms (E227K, S351Y, N394Y, T462A) are detected in the cytosol (**b**,**f**,**j**,**n**) and the GFP-CCDC6 wild type protein is mainly relocated in the cytosol (**c**,**g**,**k**,**o**). Merge images are shown in (**d**,**h**,**l**,**p**); (**C**) the myc-CCDC6 truncated isoform (1–101), tranfected in the same cells, is detected mainly in the cytosol, relocating the GFP-CCDC6 wild type protein (**b**–**d**). Hoechst nuclear staining is shown in (**A**(**a**); **B**(**a**,**e**,**i**,**m**); and **C**(**a**)). Results are representative of at least two independent experiments. Scale bar 10 mM.

By confocal microscopy, the GFP-CCDC6 wild type protein was detected with a homogeneous distribution in the cytosol and in the nucleus of HeLa Kyoto S-tag-GFP-CCDC6 cells, as expected (Figure 2A(a–d)) [1]. Conversely, the mutated isoforms of CCDC6 transiently tranfected in HeLa cells were detected mainly in the cytosol (Figure 2B(b,f,j,n)) and, most importantly, they were able to relocate the GFP-CCDC6 wild type protein predominantly in the cytosol of HeLa Kyoto S-tag-GFP-CCDC6 cells (Figure 2B(c,g,k,o)), because of the proteins heterodimerization and colocalization (Figure 2B(d,h,l,p)). 

Similarly to the mutated isoforms, the truncated isoform CCDC6^1–101^ was detected mainly in the cytosol and able to relocate the GFP-CCDC6 wild type protein (Figure 2C(a–d)) predominantly in the cytosol, as reported [1]. 

Thus, the lung-mutants of CCDC6^E227K, S351Y, N394Y and T462A^ and the truncated isoform CCDC6^1–101^, involved in CCDC6 lung fusions, can form dimers with the wild type protein and relocate the CCDC6 wild type protein predominantly out of the nucleus.

### 2.2. CCDC6 Lung-Mutants Reduce the DNA Repair Efficiency by Affecting the Homologous Recombination Process

We have tested the function of CCDC6 lung-mutants in the HR process of DNA repair by using different approaches. First, we used the 293 AJ2 human cancer cells carrying the resistance to the Blasticidin antibiotic as a DSB DNA repair reporter gene. Following the enzymatic cut induced by I-SceI in the DNA at a single chromosome locus, the HR efficiency in 293 AJ2 cells can be measured by counting the number of cells that can form colonies because of their resistance to the antibiotic selection [38].

**Figure 3:**
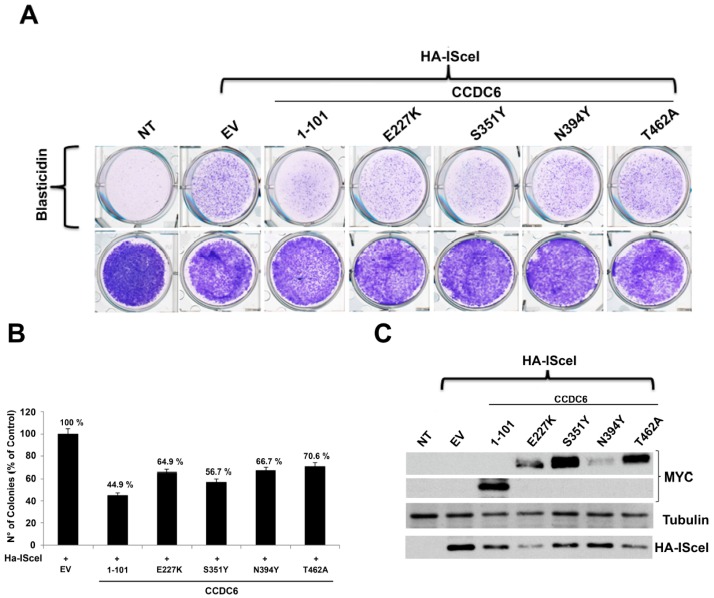
CCDC6 lung-mutants reduce the DNA repair efficiency by affecting the homologous recombination process in HR-reporter cell assay. (**A**) 293 AJ2 cells, bearing the stably integrated DNA repair construct, have been transfected with the empty vector (EV) or the myc-tagged CCDC6 mutated or truncated isoforms, as indicated. Cells were then grown in normal medium (no selection) or in the presence of Blasticidin. Colonies formed in the presence of Blasticidin were stained with crystal violet and (**B**) the colonies formed in the presence of Blasticidin were then scored and plotted, after normalization by the plating efficiency (number of colonies formed in the absence of blasticidin), percentage of I-SceI transfected cells and transfection efficiency. (**C**) The myc-CCDC6 truncated and mutated proteins overexpression was validated by western blot analysis, as indicated. Anti-HA-I-SceI and antitubulin immunoblots are shown at bottom of the panel. Results are representative of at least four independent experiments.

By using this assay the cells expressing the CCDC6 mutants or the truncated isoform showed a lower number of Blasticidin resistant colonies (CCDC6^E227K^ 64.9%, CCDC6^S351Y^ 56.7%, CCDC6^N394Y^ 66.7%, CCDC6^T462A^ 70.6%, CCDC6^1–101^ 44.9%) compared to the CCDC6 proficient cells transfected with the empty vector that correctly perform the HR DNA repair and restore the integrity of the reporter gene (Figure 3A,B). The expression of each mutated isoform of CCDC6 and of the HA-I-SceI plasmid in the transfected samples was shown in the Figure 3C (see also Figures S2 and S5).

**Figure 4:**
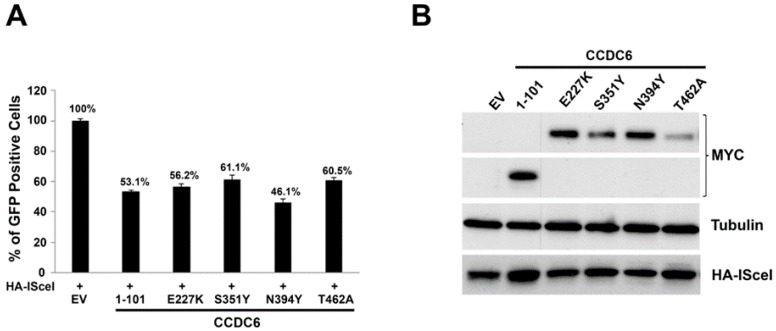
CCDC6 lung-mutants reduce the DNA repair efficiency by affecting the homologous recombination process in HR-transient cell assay. (**A**) HeLa cells were transfected with the empty vector (EV) or with the pcDNA4ToAmyc-his-CCDC6 isoforms [-CCDC6 (1–101), -CCDC6 (E227K, S351Y, N394Y, and T462A)] and with DR-GFP alone, as control, or together with I-SceI enzyme. The graph represents the percentage of GFP positive cells. Error bars indicate the standard error mean. (**B**) At western blot, the anti-myc antibody was able to detect the transiently expressed myc-CCDC6 point mutants (E227K, S351Y, N394Y, and T462A). The anti-myc antibody also detected the myc-CCDC6 (1–101) truncated isoform. Anti-HA-I-SceI and antitubulin immunoblots are shown at bottom of panel. Results are representative of at least two independent experiments.

We also analyzed the HR efficiency in the HeLa HR-GFP cell system in which the expression of GFP gene following the enzymatic cut produced by I-SceI at a single chromosome locus acts as DSB DNA repair reporter gene. The number of GFP positive cells scored by FACS analysis was lower than in control cells in all the transfected mutated isoforms of CCDC6 (CCDC6^E227K^ 56.2%, CCDC6^S351Y^ 61.1%, CCDC6^N394Y^ 46.1%, CCDC6^T462A^ 60.5%). The transfection of the truncated isoform produced, compared to the control, a lower number of GFP cells (CCDC6^1–101^ 53.1%), scored by FACS analysis as readout of HR DNA repair efficacy (Figure 4A). The expression of each mutated isoform of CCDC6 and of the HA-I-SceI plasmid in all the transfected samples was shown in the Figure 4B (see also Figures S3 and S6). Thus, as emerging by both the utilized approaches, the CCDC6 lung-mutants were able to affect the HR DNA repair, result that was reminiscent of the HR DNA repair impairment produced by the silencing of CCDC6 in the same cell systems [16]. These data suggest that the mutated isoforms of CCDC6 reported in primary NSCLC act in a dominant negative fashion with respect to the function of CCDC6 wild type protein in the HR DNA repair process. Of note, the truncated isoform of CCDC6^1–101^, involved in the oncogenic fusions to RET or ROS1 kinases in lung cancer, can form heterodimers with and relocate the wild type protein in the cytosol. Moreover, the CCDC6^1–101^ isoform, by impairing the HR DNA repair process, acts in a dominant negative fashion with respect to the CCDC6 function (second bar in Figure 3B and Figure 4A).

### 2.3. CCDC6 Lung-Mutants Induce Cisplatinum Resistance and Sensitivity to PARP-Inhibitors in NSCLC 

In order to repair the DNA DSBs, cells defective for the homologous recombination repair process switch to the non-homologous end joining repair process which involves the poly (ADP-ribose) polymerase enzymes (PARP1/2). Thus, cancer cells carrying HR DNA repair molecular alteration, like BRCA1/2 defects, are selective target of the PARP inhibition, resulting in a synthetic lethal phenotype.

**Figure 5:**
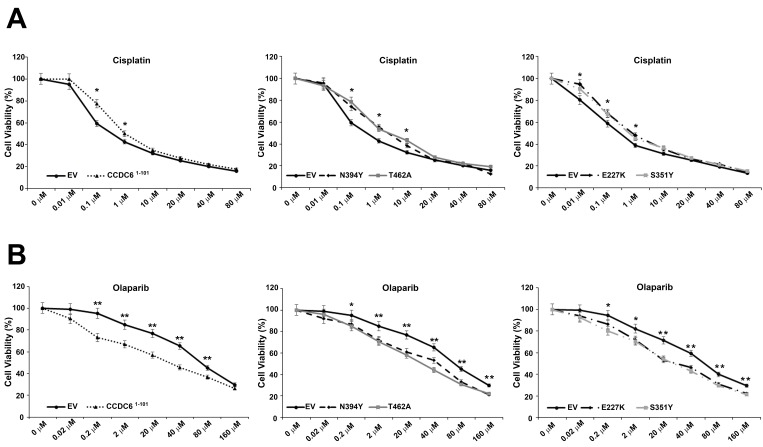

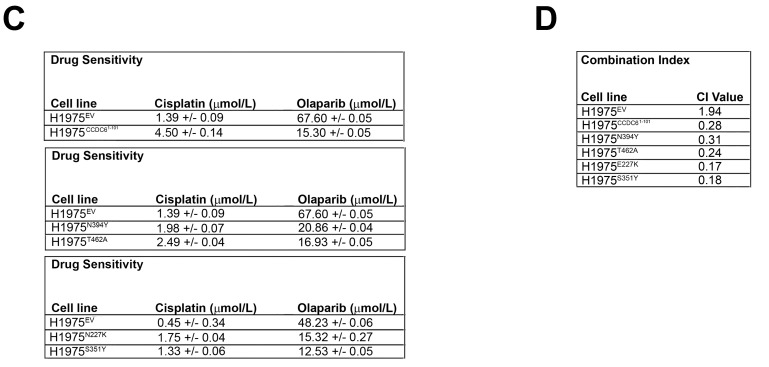
Drug sensitivity assays. Surviving fraction of NCI-H1975 (CCDC6 proficient) cells, transfected with myc-empty vector (EV), myc-CCDC6 truncated mutant (CCDC6 1–101) and myc-CCDC6 mutated isoforms (CCDC6 E227K, CCDC6 S351Y, CCDC6 N394Y, CCDC6 T462A), treated with cisplatinum (**A**) or olaparib (**B**) at the indicated doses for 144 hours are shown. Drug sensitivity to cisplatinum and olaparib in the lung cancer cells was determined by a modified 3-(4,5-dimethylthiazole-2-yl)22–5-diphenyltetrazolium bromide assay, [CellTiter 96 AQueous One Solution assay (Promega)], as 50% of survival at the inhibitory concentration (IC50) values (**C**). CI according to 1:2 concentration ratios of cisplatin and olaparib in NCI-H1975 cells transfected with the EV or with CCDC6 truncated and mutated isoforms are shown (**D**). CI < 1, CI = 1 and CI > 1 indicate synergism, additive effect and antagonism, respectively. In the diagrams, the values are presented as mean standard deviation of three independent experiments. Statistical differences were determined by two-tailed Student’s *t*-test. Statistical significance is displayed as: * *p* < 0.05; ** *p* < 0.01. The IC50 values are expressed as mean ± the standard deviation.

According to the dominant negative effect exerted by the CCDC6 lung-mutants on the HR DNA repair process, we evaluated the sensitivity of lung cancer cells to the genotoxic agent cis-platinum and to the PARPi Olaparib upon the transfection of each of the CCDC6 lung-mutant. Following the paradigm of ‘BRCAness’, the transient expression of the CCDC6 lung-mutants induced resistance to cis-platinum and sensitivity to the PARPi Olaparib in the NCI-H1975 lung cancer cells (Figure 5). This cell line has been chosen since it has been already well characterized for CCDC6 expression and drugs sensitivity upon the CCDC6 silencing [16]. The IC50 values of the NCI-H1975 lung cancer cells, expressing the different CCDC6 lung-mutants and treated with a range doses of cis-platinum, were higher than in control cells indicating an induction of cis-platinum resistance (Figure 5A,C). Nevertheless, the IC50 values of the NCI-H1975 lung cancer cells, expressing the different CCDC6 lung-mutants and treated with a range doses of Olaparib, resulted lower than in the control cells, indicating an induction of sensitivity to Olaparib (Figure 5B,C). Similarly, the IC50 values of the NCI-H1975 lung cancer cells expressing the truncated isoform of CCDC6^1–101^ indicated cis-platinum resistance and Olaparib sensitivity compared to control cells (Figure 5A–C), according to the dominant negative activity exerted by the CCDC6^1–101^ truncated isoform with respect to the CCDC6 wild type function in HR DNA repair process, as reported in the previous paragraph.

Interestingly, the combination of cis-platinum and Olaparib (at ratio of 1:2) was able to overcome the cis-platinum resistance in the NCI-H1975 lung cancer cells transfected with the mutated and truncated CCDC6 isoforms, leading to a synergistic effect of the two drugs (CI < 1), as previously reported in the same cells upon the CCDC6 silencing (Figure 5D) [16]. 

## 3. Discussion

Cells activate powerful DNA cell cycle checkpoints and DNA repair proteins to recover from the genotoxic injuries [39,40,41,42,43,44]. The overall importance of the cell cycle checkpoints and DNA damage repair (DDR) proteins in maintaining genomic integrity is highlighted by the observation that the genes involved in the DDR process are often lost, mutated, or silenced in cancer cells [45,46,47,48,49]. Because of its role in the surveillance of DNA integrity, CCDC6 has been proposed as a tumor suppressor gene [4,50].

Indeed, low levels of expression of CCDC6 protein and several CCDC6 fusions have been reported in many tumor types [12,13,14,15,16,17,18,19,20,21,22,23]. Low levels of CCDC6 protein have been reported in about 30% of NSCLC and correlated with prognosis [16]. Moreover, CCDC6 has been found fused to RET and ROS1 genes in about 1% of NSCLC [30,31,32,33,34].

Recently, almost 135 molecular alterations in CCDC6 gene have been identified so far in different tumor types, consisting of missense mutations (13.79 %), nonsense mutations (2.30 %) and either insertion or deletion (2.3%), all distributed along the entire sequence of the gene with no evident hot spots of mutation (https://cancer.sanger.ac.uk/cosmic) [35]. The majority of the mutations reported for CCDC6 consists in the change of a single amino acid.

A systematic study to functionally classify CCDC6 gene mutations or rearrangements in primary tumors is still missing. Here we show that the mutants of CCDC6 identified so far in NSCLC can form heterodimers with the wild type CCDC6 protein and act as dominant negative of the CCDC6 function in the repair of DNA double strand breaks, inducing cis-platinum resistance and PARPi sensitivity. We also show for the first time that the first 101 aa of CCDC6 involved in the CCDC6 fusions reported in NSCLC, can functionally impair the HR DNA repair process and affect cancer cell sensitivity to selected drugs.

The effect exerted by the CCDC6 mutants and CCDC6 truncation observed in CCDC6 proficient cells is similar to the effect obtained by the CCDC6 silencing in the same cell systems indicating a dominant negative role. Besides of experimental variability, the extension of the formation of the heterodimers between the CCDC6 lung mutants and CCDC6 wild type protein could be different depending on the affinity of the interaction, on the stability of the different mutants and/or on the intracellular distribution of the CCDC6 mutants. However, the dominant negative function of the CCDC6 mutants could rely on reduction of the nuclear amount of the CCDC6 wild type protein upon the formation of heterodimers in the cytosol. The biochemical mechanisms for the nuclear reduction induced by the CCDC6 mutated isoforms (1–101, E227K, S351Y, N394Y, and T462A) is in need of further investigation. It can be postulated that the CCDC6 mutations identified in NSCLC patients can affect post-translational modifications of the CCDC6 protein, meaning either the phosphorylation that drives the CCDC6 localization in the nucleus or the stabilization of the protein in the cytosol [1]. The heterocomplex in which the mutated isoforms of CCDC6 colocalize with the wild type protein mainly in the cytosol might feature intracellular structures in which the wild type protein could be dynamically trapped by the mutated isoform during the process of protein maturation. 

**Figure 6:**
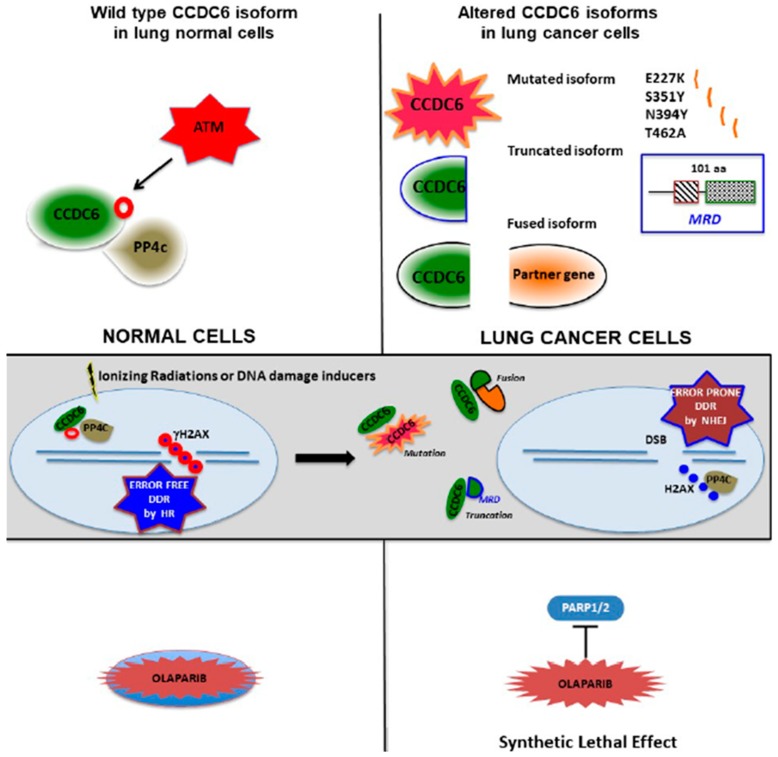
Sketch of the impaired functional mechanisms in lung cancer cells carrying CCDC6 molecular alterations. Upper section, left side: the wild type isoform of CCDC6 protein is a target of the ATM kinase and forms a protein complex with the phosphatase PP4c; right side: the molecular alterations of CCDC6 protein in NSCLC consist in missense mutations, truncation at aa 101 or fusion with a partner gene. Middle section, left side: in normal cells, DNA damage inducers cause the phosphorylation of CCDC6 (red ring) upon the activation of ATM and the formation of a CCDC6 complex with the phosphatase PP4c, leading to a stable phosphorylation of histone H2AX (red ring) at the site of double strand breaks (DSB) of DNA which prompts an error-free DNA damage repair (DDR) based on the homologous recombination (HR) process; right side: in lung cancer cells, the presence of any of the CCDC6 alterations can induce the formation of protein extranuclear heterocomplex between the CCDC6 altered isoform and the CCDC6 wild type protein, excluding the CCDC6 wild type protein from its interaction with the phosphatase PP4c. In these circumstances, the released PP4c phosphatase induces the dephosphorylation of the histone H2AX at the site of double strand breaks (DSB) of DNA, causing a faster resolution of the repair protein complexes and the activation of an error prone DNA damage repair (DDR) based on the non-homologous end joining (NHEJ) process. Lower section, the treatment with Parp1/2 inhibitor Olaparib is not effective in normal cells (left side) while is efficacious in lung cancer cells carrying the different isoforms of CCDC6 (right side) because of a synthetic lethal effect.

CCDC6 interacts with proteins involved in the DNA damage response and repair complexes including ATM, PP4c, and BAP1 [2,9,15]. It is reasonable that the sequestration of CCDC6 wild type protein out of the nucleus, as well as the loss of CCDC6 protein expression, might induce the release of PP4c phosphatase leading to the dephosphorilation of H2AX^S139^ and to the resolution of the DNA repairing foci. Thus, cancer cells that carry the CCDC6 missense mutations, truncation, or fusions are prone to accumulate errors in DNA repair process with a genome instability that can promote tumor progression, leading to radio and chemoresistance. We summarize the characteristic of CCDC6 molecular alterations identified in lung cancer patients in the diagram of Figure 6, proposing a model of their function in cancer cells.

In this work, we demonstrated that the transient expression of the CCDC6 lung mutant or truncated isoforms in lung cancer cells determined a trend of cis-platinum resistance and Olaparib sensitivity as previously observed in the same cells stably silenced for CCDC6 [16]. 

Platinum salts represent the gold standard treatment for NSCLC patients, even if their administration is limited because of high toxicity and high rate of resistance. Several mechanisms involving DNA damage repair (DDR) defects have been proposed to account for the resistance to platinum agents and radiations [45,46,47,48,49]. Molecular alterations affecting the DDR genes, such as ERCC1/2 and BRCA1/2, are emerging as predictors of cancer response to conventional chemo-therapeutics, and their evaluation is now proposed in several clinical trials including the NSCLC with the aim to correlate the defects of selected DDR genes with the efficacy of new targeted therapies [11,51,52,53,54,55,56,57,58,59,60]. Our study indicates that the molecular alterations of CCDC6 by impairing the CCDC6 nuclear function may act as ‘BRCA like’ alterations, making cancer cells selective target of PARPi Olaparib which might help to overcome the resistance to the conventional anticancer therapy in NSCLC patients. The PARPi treatment might also be considered in the ‘maintenance therapy’ for NSCLC patients carriers of CCDC6 or other DDR genes molecular alterations. 

Currently, CCDC6 alterations are identified in the clinic of NSCLC because of the CCDC6 fusions to the RET and ROS1 kinases. Furthermore, the use of kinase inhibitors of EGFR [61], ALK [62], and RET [63], has been beneficial in about 10–15%, 5–7%, and 1.9% of NSCLC patients, respectively. Our findings suggest that the NSCLC patients carrying the CCDC6 fusions, beside the kinase inhibitors, might also benefit of the inclusion of PARPi in order to prevent cancer cell resistance.

Although the role of CCDC6 in NSCLC should be further investigated, the results discussed here clearly suggest that the evaluation of CCDC6 protein by IHC, besides considering the levels of protein expression, should also consider the evaluation of protein distribution in the cancer cells (nucleus > cytosol) which might be consequential of CCDC6 molecular alterations. A ‘basket study’ based on a wide IHC analysis is now in progress and is meant to group patients with different CCDC6 expression and intracellular distribution (Merolla F. et al., in preparation). In conclusion, the low levels of nuclear CCDC6 protein detected by IHC might disclose those cases in which the analysis of CCDC6 mutations or rearrangements cannot be explored as first assessment.

Thus, in future clinical trials, CCDC6 protein expression and distribution should be assayed by IHC analysis in addition to the molecular screen of DDR genes defects and to an accurate estimate of the HRD, in order to select NSCLC patients that could benefit of radio and chemotherapy when combined with PARP inhibitors [64,65,66]. 

Additional molecular alterations in CCDC6 gene, recently reported in different primary tumors, might imply similar effects on cancer cell resistance to conventional anti-cancer therapy and possibly predict the efficacy of the combined treatment with PARPi in further patients. 

## 4. Materials and Methods

### 4.1. Cell Lines, Drugs, and Chemicals

The human cell line NCI-H1975 was provided by Professor Fortunato Ciardiello and was cultured in RPMI 1640 (Gibco, Paisley, UK), supplemented with 10% fetal bovine serum (Gibco, Paisley, UK), 1% penicillin/streptomycin (Gibco, Paisley, UK). No RET/PTC1 fusion or CCDC6 mutations have been reported in this cell line [31]. HeLa Kyoto S-tag-GFP-CCDC6 cells were generated and kindly provided by Ina Poser and Anthony Hyman [6,67], and were grown in DMEM (high glucose), 10% FCS, 1% penicillin/streptomycin (Gibco, Paisley, UK). 293 AJ2 cells were obtained by Professor Spiros Linardopoulos and were grown in Dulbecco’s modified Eagle’s medium (DMEM, Gibco, Paisley, UK) supplemented with 10% fetal calf serum (FCS), 2.4 mM L-glutamine, 100 U/mL penicillin, and 100 µg/ml streptomycin. Cycloheximide was obtained from SIGMA-Aldrich, Inc. (St. Louis, MO, USA). Olaparib was provided by SelleckChem (AZD2281). Hoechst 33258 and cisplatinum were from SIGMA-Aldrich, Inc (St. Louis, MO, USA). 

### 4.2. Plasmids and Transfection

PcDNA4ToA-CCDC6 wt and pcDNA4ToA-CCDC6 (1–101) plasmids were generated and transfected with FuGene HD (Promega, Madison, WI, USA) as described elsewhere [1]. From the pcDNA4ToAmyc-his-CCDC6 wild type template several myc-CCDC6 mutants (E227K, S351Y, N394Y, and T462A) were created using the Quick Change Site Directed Mutagenesis Kit (Agilent, Santa Clara, CA, USA). The oligo sequences are reported in the Table S1. Lipofectamine 2000 (Invitrogen, Carls Bad, CA, USA) was used for transient transfections with the DR-GFP plasmid. The DR-GFP reporter plasmid is based on a construct developed by M. Jasin [68] and contains two mutated GFP genes separated by a puromycin drug selection marker. Transfection of 293 AJ2 cells was carried out using Lipofectamine 2000 (Invitrogen) according to the manufacturer’s protocol.

### 4.3. Protein Extraction and Western Blot Analysis

Total cell extracts were prepared with RIPA Buffer (50 mM Tris-HCl pH 7.5, 150 mM NaCl, 1% Triton X-100, 0.5% Na Deoxycholate, 0.1% SDS) and a protease inhibitor cocktail (Roche, Basilea, Switzerland). Immunoblotting experiments were carried out according to standard procedures and visualized using the ECL chemiluminescence system (Amersham, Little Chalfont, UK/Pharmacia Biotech, Milano, Italy). Anti-myc monoclonal antibody (clone 9E10_sc40) was from Santa Cruz Biotechnology, Inc, Dallas, TX, USA; anti-GFP and anti-HA were from Covance, Inc, Princeton, New Jersey, USA; anti-CCDC6 (ab 56353) was from ABCAM plc, Cambridge, UK; anti-tubulin was from SIGMA-Aldrich, Inc. 

### 4.4. Immunofluorescence Staining

Transfected and control cells were fixed with 4% paraformaldehyde and treated with phosphate-buffered saline (PBS)/0.25% Triton X-100. The immunofluorescence staining was performed with the anti-myc primary antibody, followed by washing with PBS and incubation for 30 min at room temperature with secondary anti-mouse antibody (Jackson Laboratory, please add address). Hoechst staining was utilized for nuclear visualization. 

### 4.5. HR Reporter Cell Assay

293 AJ2 cells were transfected with pcDNA4ToAmyc-his-CCDC6 (1–101), -CCDC6 (E227K, S351Y, N394Y, and T462A) point mutants and empty vector (EV) and plated for colony forming assays [38]. 

### 4.6. HR Transient Assay

HeLa cells transfected with pcDNA4ToAmyc-his-CCDC6 (1–101), -CCDC6 (E227K, S351Y, N394Y, and T462A) point mutants and empty vector (EV) were plated in a 12 well-plate and transfected with the DR-GFP reporter alone (as negative control), or together with the I-SceI gene. Wild type GFP was used as control for transfection efficiency. After 48 h cells were collected and analyzed by FACS analysis with BD Accuri C6 Flow Cytometer (BD Bioscience, Franklin Lakes, NJ, USA). The DR-GFP and the HA-SceI plasmids were kindly provided by Professor Vittorio Enrico Avvedimento.

### 4.7. Sensitivity Test and Design for Drug Combination

A modified 3-(4,5-dimethylthiazole-2-yl)-2-5-diphenyltetrazolium bromide assay, CellTiter 96 AQueous One Solution assay (Promega, Madison, WI, USA), was utilized to test the drugs antiproliferative activity as expression of 50% of cells survival at the inhibitory concentration (IC50) values. After plating the cells at a density of 2000 cells per well, each drug was added for 144 h. Each assay was performed in quintuplicate and IC50 values were expressed as mean +/− standard deviation. After the treatment with different drugs in combination the results were analyzed according to the method of Chou and Talaly by using the CalcuSyn software program [69]. The derived combination index (CI) represented a quantitative measure of the grade of interaction between different drugs. A CI value of unity denotes additive activity while CI > 1 denotes antagonism, and CI < 1 denotes synergy between agents.

## 5. Conclusions

In conclusion, the molecular alterations involving CCDC6 gene product might result both in reduced levels of CCDC6 protein or in a redistribution of the intracellular compartmentalization of the protein. Thus, the CCDC6 characterization in lung cancer patients may define indications to select group of patients who could benefit of PARP-inhibitors treatment in combination with standard therapies.

## Supplementary Materials

The following are available online at https://www.mdpi.com/2072-6694/12/1/44/s1, Table S1: Oligi sequence for CCDC6 mutagenesis, Figure S1: With reference to Figure 1, the whole films with all the bands and molecular weight marker are shown, Figure S2: With reference to Figure 3, the whole films with all the bands and molecular weight marker are shown, Figure S3: With reference to Figure 4, the whole films with all the bands and molecular weight marker are shown, Figure S4: Densitometric analysis has been performed by Image J Software and histograms represent the relative protein levels of MYC CCDC6 normalized to GFP (upper graph) or Tubulin (lower graph), expressed as relative intensity compared to controls, Figure S5: Densitometric analysis has been performed by Image J Software and histograms represent the relative protein levels of MYC CCDC6 (upper graph) and HA- ISceI (lower graph) normalized to Tubulin and expressed as relative intensity compared to non-transfected controls, Figure S6: Densitometric analysis has been performed by Image J Software and histograms represent the relative protein levels of MYC CCDC6 (upper graph) and HA- ISceI (lower graph) normalized to tubulin and expressed as relative intensity compared to control (empty vector, EV).
