# Supplementary material for: NSCLC Mutated Isoforms of CCDC6 Affect the Intracellular Distribution of the Wild Type Protein Promoting Cisplatinum Resistance and PARP Inhibitors Sensitivity in Lung Cancer Cells
Source: Cancers (Basel). 2019 Dec 21;12(1):44. doi: 10.3390/cancers12010044 (PMC7016757; doi:10.3390/cancers12010044)
Supplement: Supplementary file 1 [file cancers-12-00044-s001.pdf]

Primer	Sequence
**E227K Fw**	atggataagcttgaagctaaaaagcgaatcctgcagg
**E227K RV**	cctgcaggattcgctttttagcttcaagcttatccat
**S351Y Fw**	aggattaagacctcgcactgtgtatagcccgatccct
**S351Y Rv**	agggatcgggctatacacagtgcgaggtcttaatcct
**N394Y Fw**	gagctggaatgtcttattactattccccgggtctt
**N394Y Rv**	aagacccggggaatagtaataagacattccagctc
**T462A Fw**	atgttgcgaaggagcaggctgcgaggtgg
**T462A Rv**	ccacctcgcagcctgctccttcgcaacat

Table S1: Oligi sequence for CCDC6 mutagenesis

Figure S1

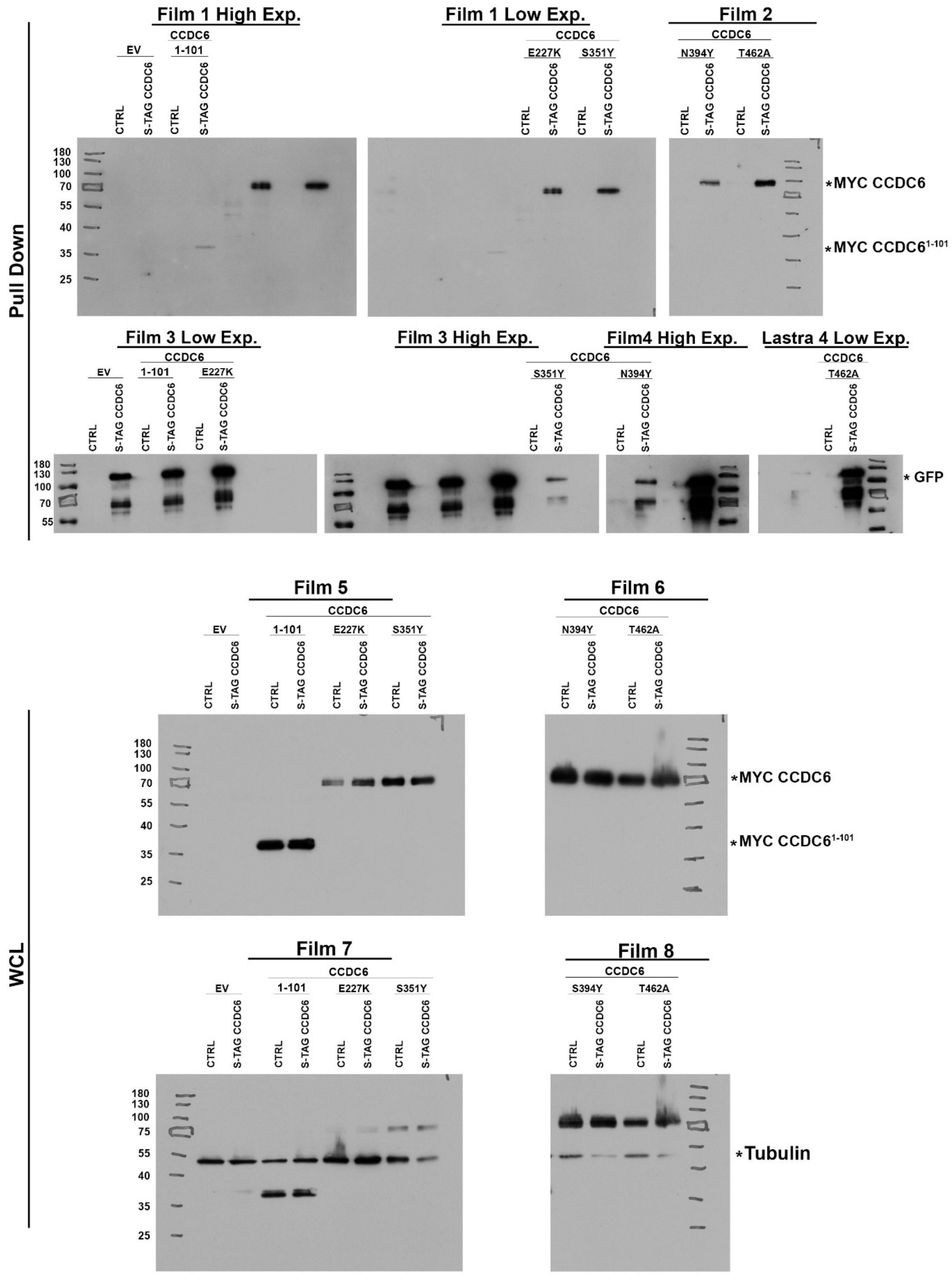

Figure S1: With reference to figure 1, the whole films with all the bands and molecular weight marker are shown.

Figure S2

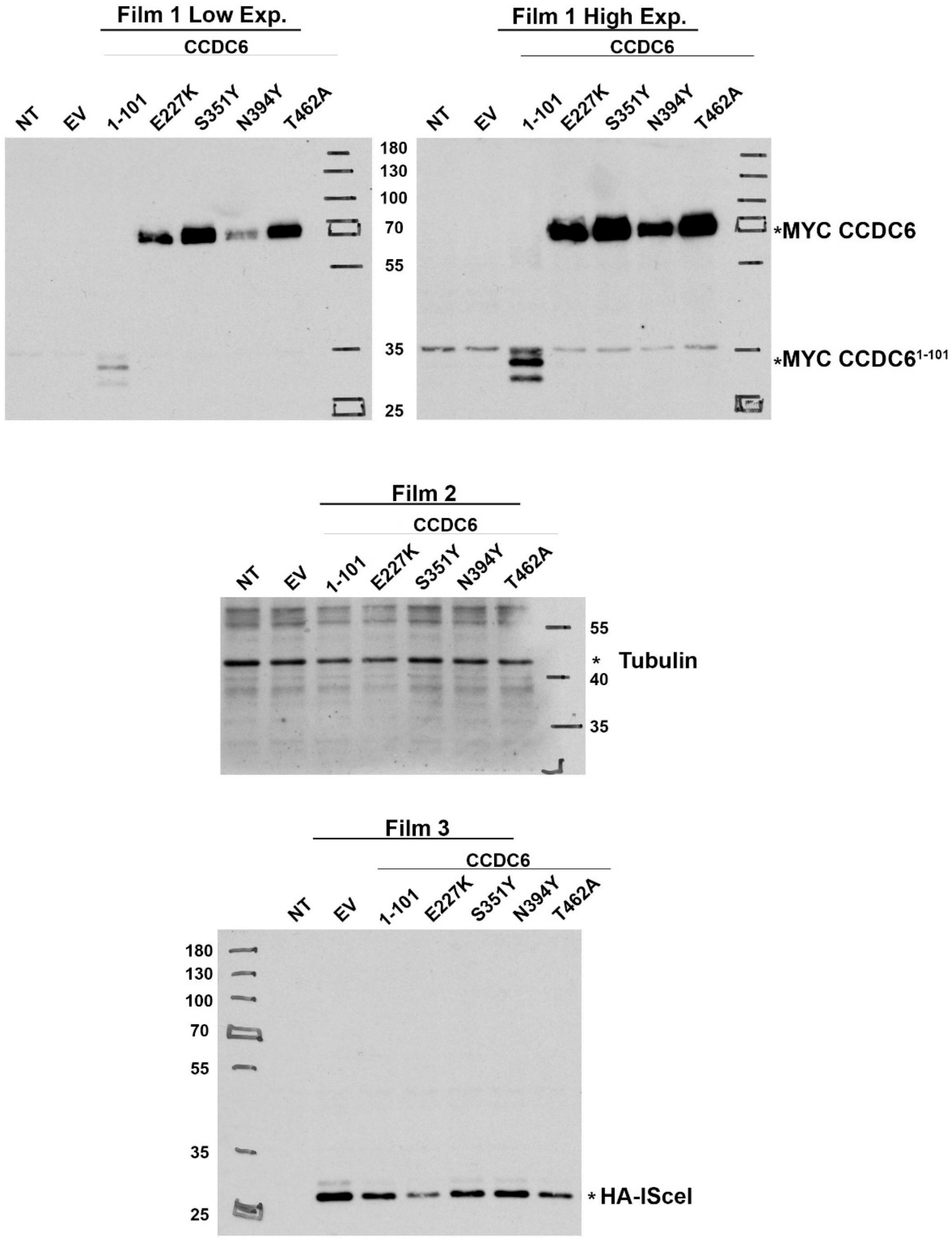

Figure S2: With reference to figure 3, the whole films with all the bands and molecular weight marker are shown.

Figure S3

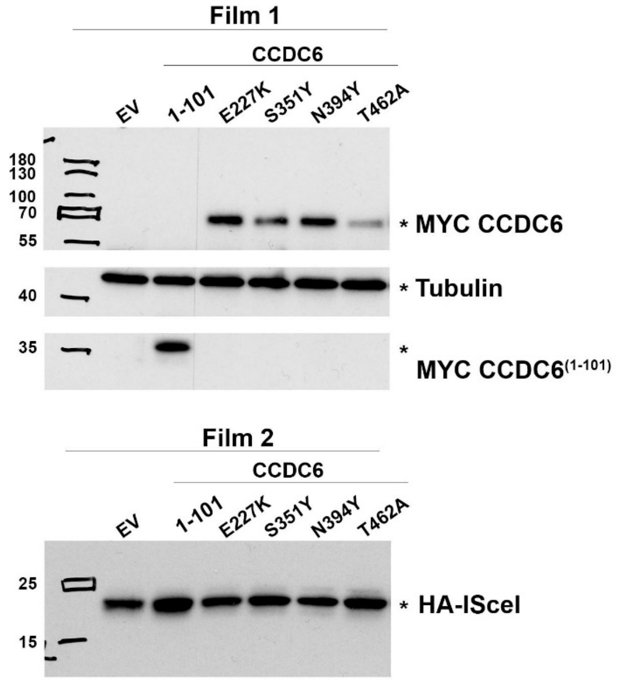

Figure S3: With reference to figure 4, the whole films with all the bands and molecular weight marker are shown.

Figure S4

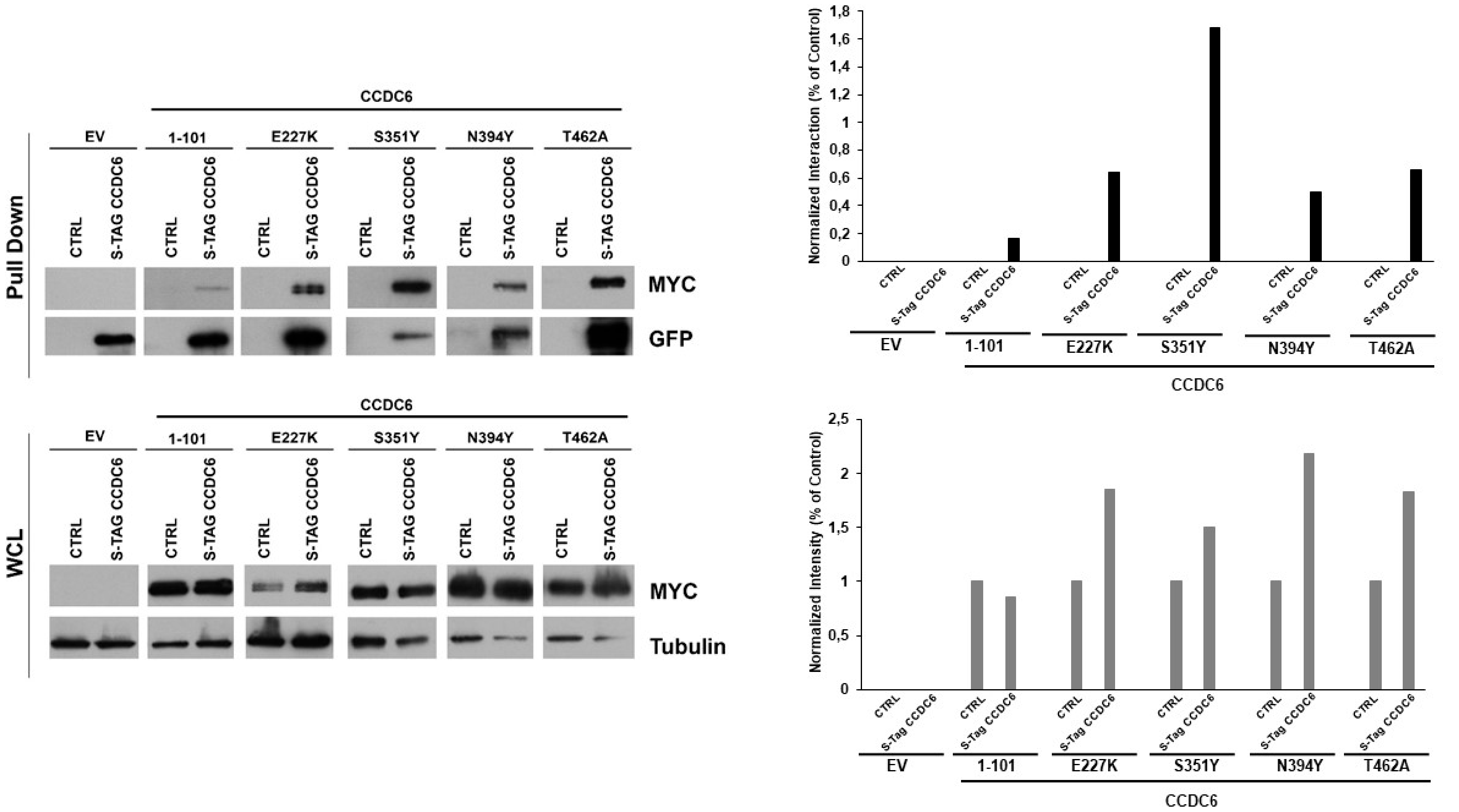

Figure S4: Densitometric analysis has been performed by Image J Software and histograms represent the relative protein levels of MYC CCDC6 normalized to GFP (upper graph) or Tubulin (lower graph), expressed as relative intensity compared to controls.

Figure S5

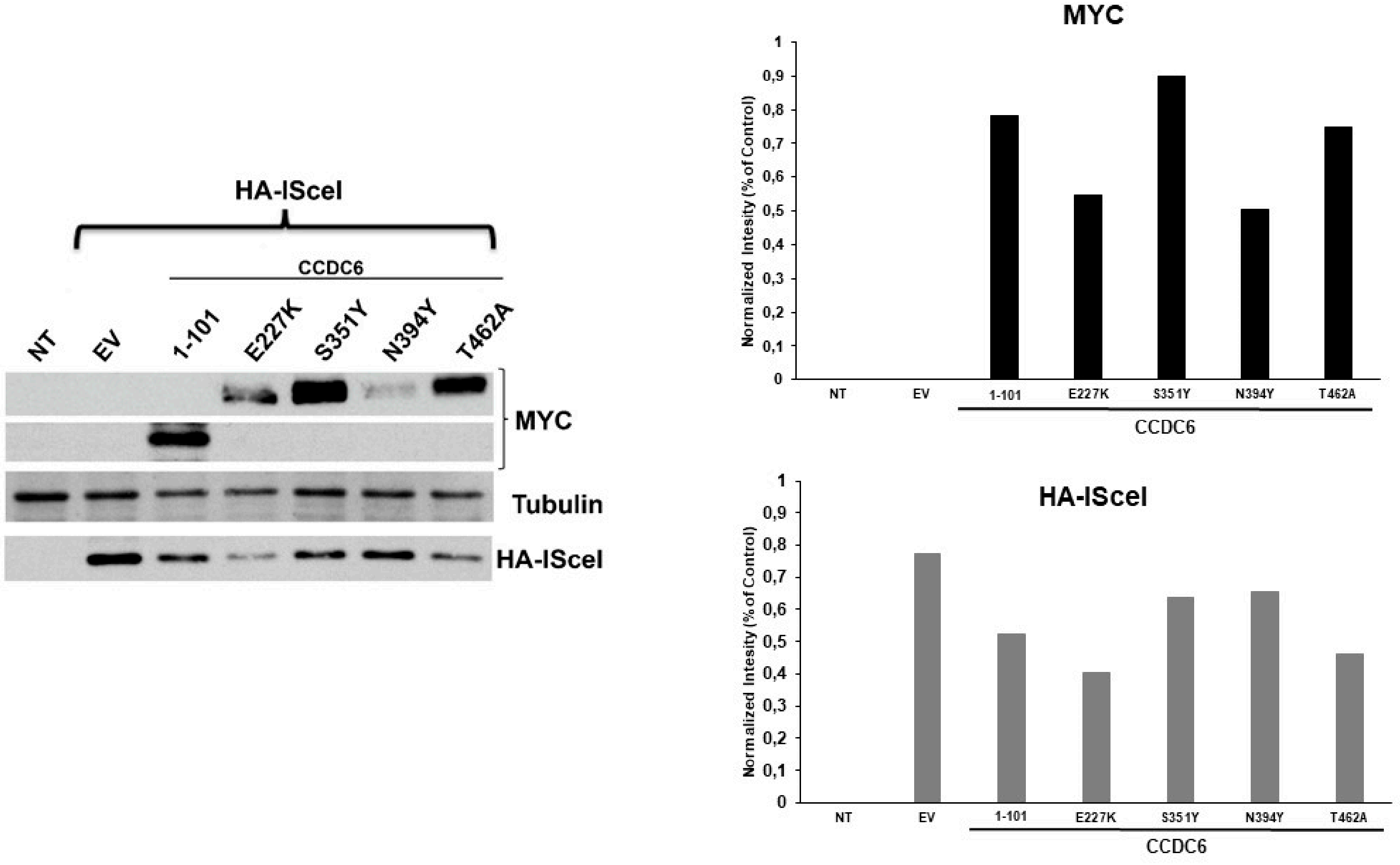

Figure S5: Densitometric analysis has been performed by Image J Software and histograms represent the relative protein levels of MYC CCDC6 (upper graph) and HA- ISceI (lower graph) normalized to Tubulin and expressed as relative intensity compared to non-trasfected controls.

Figure S6

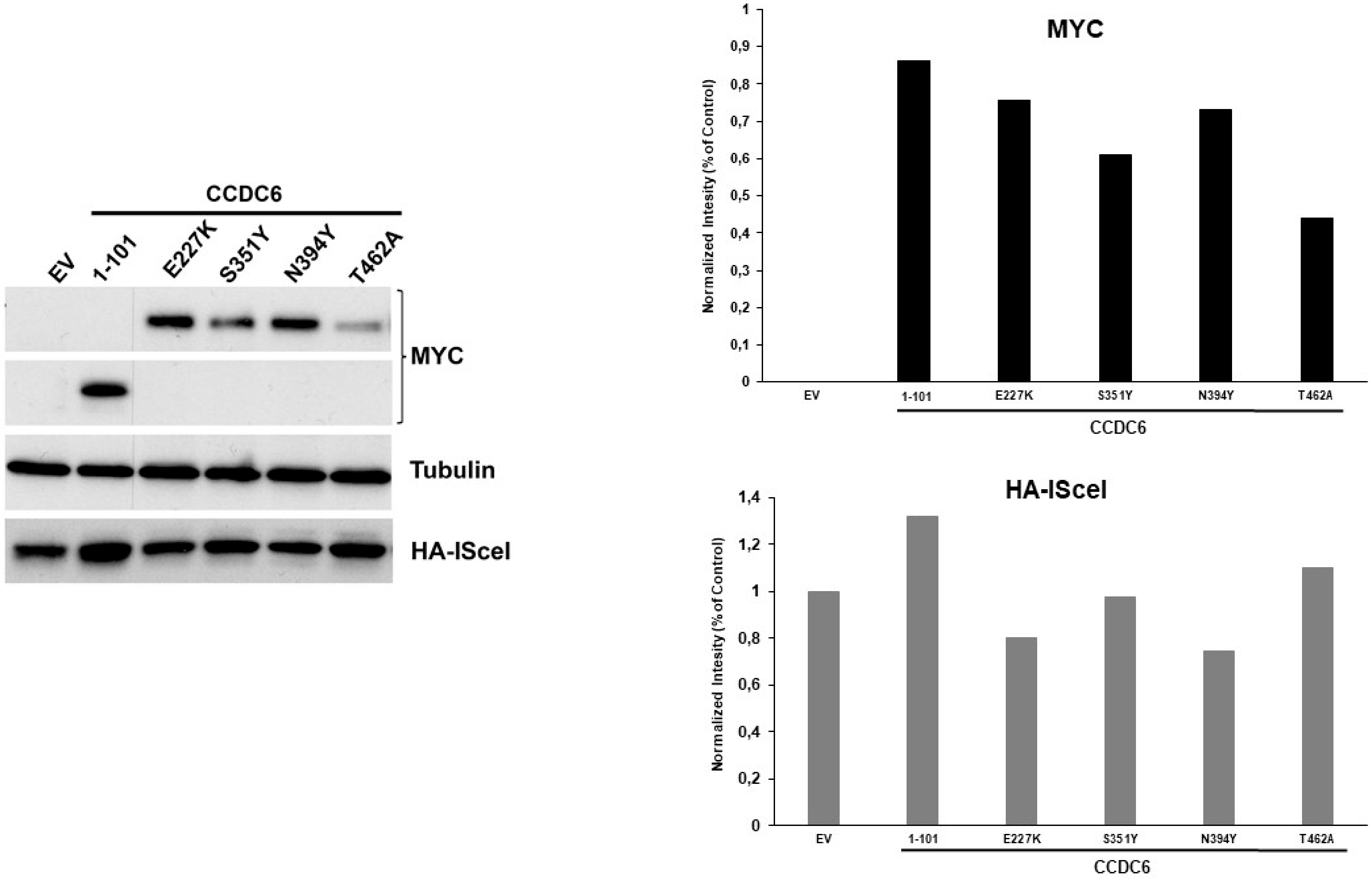

Figure S6: Densitometric analysis has been performed by Image J Software and histograms represent the relative protein levels

of MYC CCDC6 (upper graph) and HA- IScel (lower graph) normalized to Tubulin and expressed as relative intensity compared to control (empty vector, EV).
